# Satisfaction, discomfort, obligations, and concerns in population-based breast cancer screening: cross-sectional study in a Danish population

**DOI:** 10.1186/s12913-017-2438-2

**Published:** 2017-07-14

**Authors:** Pernille Gabel, Mette Bach Larsen, Pernille Bjørnholt Nielsen, Dorte Brandt Svendstrup, Berit Andersen

**Affiliations:** 1 Department of Public Health Programmes, Regional Hospital Randers, Skovlyvej 15, DK-8930 Randers NØ, Denmark; 2 DEFACTUM, Central Denmark Region Aarhus, Olof Palmes Alle 15, DK 8200 Aarhus N, Denmark

**Keywords:** Breast neoplasms, Early detection of cancer, Patient satisfaction, Mass screening, Cross-sectional studies

## Abstract

**Background:**

Potential barriers to breast cancer screening adherence include patient satisfaction, as well as pain, feeling obliged to participate, and other concerns that might compromise the level of satisfaction.

The present study aimed to assess the overall satisfaction of Danish citizens with their breast cancer screening experiences, as well as their level of discomfort, concerns, and feelings of obligation to participate. Furthermore, we analyzed the associations between overall satisfaction and the remaining outcomes.

**Methods:**

Questionnaires were mailed to 3000 women in the Central Denmark Region who received screening examination results in the fall of 2013. The questionnaire assessed satisfaction (overall, telephone hot-line, and web-based self-service), discomfort (pain and boundaries of modesty), concerns (at invitation, while waiting for results, and after receiving results), and feelings of obligation to participate. Background information was retrieved from Statistics Denmark.

Pearson’s chi-square test was used to test differences in outcomes and demographic characteristic distributions between respondents and non-respondents and highly satisfied vs. less satisfied participants. Prevalence ratios (PR) with 95% CI were assessed using Poisson regression with robust variance, to estimate associations between satisfaction and the remaining outcomes.

**Results:**

Among the participants, 70.3% and 29.4%, respectively, reported really good and good impressions of the screening program. Lower satisfaction was associated with feeling pain (prevalence ratio (PR), 0.82), feeling that modesty boundaries were transgressed (PR, 0.79), experiencing screening-induced concerns (PR, 0.84), and feeling obliged to participate (PR, 0.96). Of the participants, 36.2% and 12.9%, respectively, felt very much and moderately obliged to participate. A total of 72.6% reported no screening-induced concerns, including 73.3% of those with negative screening results and 38.1% of those with positive screening results.

**Conclusions:**

Overall satisfaction with breast cancer screening was very high, but discomfort, feelings of obligation, and concerns were associated with lower satisfaction levels. A continuing focus on high service in breast cancer screening is important for achieving the highest benefit from the program. This includes initiatives to employ the least painful techniques, to respect the patients’ modesty as much as possible, and to deliver fast screening results and thus minimize concerns among women awaiting results.

**Electronic supplementary material:**

The online version of this article (doi:10.1186/s12913-017-2438-2) contains supplementary material, which is available to authorized users.

## Background

Among women worldwide, breast cancer is the most prevalent cancer and the leading cause of cancer-related death [1]. Early breast cancer diagnosis and optimal treatment can reduce mortality [2]. Breast cancer screening aims to detect the disease at an early stage to improve prognosis, and can reportedly lead to a 15–20% reduction in breast cancer mortality [3, 4]. Therefore, many Western countries have introduced population-based breast cancer screening programs [5], although there remains debate regarding the benefits and harms [6, 7].

To achieve mortality reduction with breast cancer screening, the targeted population must adhere to the screening guidelines [8]. Several factors are reportedly associated with following recommendations for repeated mammographies—including satisfaction with clinic service and/or healthcare providers (e.g., communication and accessibility), low physical discomfort (e.g., experiencing pain), and low psychological distress (e.g., experiencing concerns or embarrassment) [9, 10].

Since 2008, under Danish law, all Danish women of 50–69 years of age have been offered biennial breast cancer screening free of charge [11]. The participation rate in the Danish national breast cancer screening program reached 84.3% in 2012–2013 [12]. In order to maintain this high participation rate, it is important to ensure continuing good user experiences in the screening program.

It has been hypothesized by Ploug et al. that the Danish breast cancer screening program is organized in a manner that makes women feel obliged to participate, which is in opposition to the right of free choice [13]. However, this implied obligation to participate has never been examined within the Danish breast cancer screening program.

The present study aimed to assess the overall satisfaction with breast cancer screening in a Danish population, including specific assessment of the level of discomfort, concerns, and feelings of obligation to participate. We further intended to analyze associations between overall satisfaction and demographic characteristics, discomfort, concerns, and feelings of being obligated to participate.

## Methods

### Study design and setting

We designed a cross-sectional study using survey and register data. In accordance with Danish law, the study was approved by the Danish Data Protection Agency (Journal Number: 2007–58-0010/Case number: 1–16–02-423-13).

This study was performed in the Central Denmark Region (CDR), which comprises both rural and urban areas, and includes the city of Aarhus, the second largest city in Denmark. The CDR has approximately 1.3 million residents (October 1, 2013) corresponding to approximately 23% of the total Danish population. These residents included 159,726 women 50–69 years of age [14].

The invitation to participate in breast cancer screening includes a scheduled appointment for a screening mammography at one of five screening units in the CDR. To unsubscribe from the program or change their appointment, women can contact the Department of Public Health Programmes by telephone or e-mail, or by using a web-based self-service. If a woman does not attend the scheduled screening, she will receive one reminder without another pre-booked appointment. Information about the screening program is available in an information booklet. The booklet is available online, and is also sent to the women once with their first invitation to screening. The booklet contains information on breast cancer, breast cancer screening, and advantages and disadvantages of screening participation.

One of the five screening units is situated at a hospital, and the other four are located outside of the hospital, for example, in a shopping or healthcare center. Participants’ average distance to the chosen screening unit is 23.2 km (range, 0–118.3 km) [15]. Mammographies are performed by specially trained healthcare assistants. The process is streamlined to enable assistants to screen 10–12 women per hour at each mammomat. Two images are taken of each breast (cranio-caudal and mediolateral oblique), which are later independently evaluated by two screening radiologists [11].

Women receive their screening results within 2 weeks. If evaluation of the screening images cannot rule out cancer, an appointment is scheduled for further diagnostic procedures at the nearest hospital within six calendar days from the date the results are released. If the screening reveals no sign of cancer, the woman is informed that she will be invited again in 2 years if still within the screening age range, and that she should seek medical advice in the event of any breast cancer symptoms regardless of the time since last screening mammography. The women’s general practitioners are simultaneously informed of the screening results.

### Participants

Women in the CDR who received breast cancer screening results during weeks 42 to 49 of 2013 were eligible for inclusion in this study. All Danish residents are listed in the daily updated Civil Registration System, which includes unique civil registration numbers (CRNs), names, and postal addresses [16], and is linked to the CDR’s administrative breast cancer screening system. From the administrative system, eligible women were identified once every 2 weeks, and the study included a random sample of 150 women from each screening unit. Women were randomly selected using the RAND-function in Microsoft Office Excel (2003 version): after assigning the women a random number between 0 and 1, the women were ordered according to the random numbers. The first 150 women from each screening unit were chosen to receive a questionnaire.

**Fig. 1:**
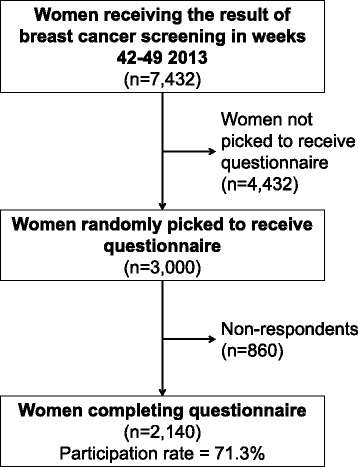
Flow chart depicting the inclusion in the study

The included women were sent a questionnaire along with a description of the survey. Women were asked to fill out a questionnaire on their experiences in their recent breast cancer screening participation for quality assurance purposes. Hence the women received the questionnaire and survey invitation within 9 days after they received their screening results. Participating women could complete the questionnaire and return it to the study office by ordinary mail in a pre-addressed pre-stamped envelope, or they could complete it online using a unique code provided in the accompanying letter. No reminders were issued. Women were included in the study if they returned the questionnaire no later than December 23, 2013 (Fig. 1).

### Questionnaire content and development

The questionnaire was in Danish, and was a revised and extended version of a standard questionnaire that is regularly used for quality assurance in hospital departments in Denmark [17]. It comprised standard questions regarding satisfaction, supplemented with questions relating to discomfort, feelings of obligations, and concerns specific to the breast cancer screening procedure (Additional file 1). The questions were tested in the target population at a regional screening unit—first in 2010, when the questions on satisfaction and discomfort were initially tested in 19 selected women, and next in 2013, when the questions on obligations and concerns were added and tested in a group of 10 women. Participants were asked to complete the questionnaire, and were then individually interviewed about their understanding of the questions and their relevance. Both procedures were conducted using semi structured interviews, and resulted in the identification of minor misunderstandings, that prompted corrections in the questionnaire. The final questionnaire included a total of 50 items on different aspects of satisfaction in regards to service quality, expectations, program logistics, parking facilities, waiting times etc. A total of 10 items were used for analyses in the present study. These 10 items are described in the Data section.

### Data

The presently analyzed data included information from the screening program’s administrative system in the CDR, registry data from Statistics Denmark, and survey data. Age at the time of receiving the screening result was retrieved from the administrative system, and was categorized into four groups: 50–54, 55–59, 60–64, and 65–69 years of age. Demographic data were collected from Statistics Denmark [18]. Ethnicity was classified as Danish, immigrant from western countries, or immigrant from non-western countries utilizing definitions provided by Statistics Denmark. Educational level was classified as low (≤10 years), medium (10–15 years), or high (>15 years) according to the UNESCO classification of education [19]. Marital status was recorded as married/cohabitating or single.

Overall impression is a global item covering the women’s feelings about the screening program. Impressions are thought to cover both expected and percieved quality of the program, and according to Mohamed et al., perceived quality is related to patient satisfaction [20]. Thus, in our study satisfaction with breast cancer screening was assessed based on three questionnaire items: *“What was your overall impression of the entire mammography screening process (from receiving the invitation until response letter was received)?”* (item 41), *“What was your overall impression of the service offered by phone?”* (item 25), and *“What was your overall impression of the self-service facility offered?”* (item 15). Only the first item was used to analyze associations.

Very few respondents gave low scores on the item on overall impression of the screening process (satisfaction) (Table 2). Thus, for our analyses, the response *“Excellent”* was coded as highly satisfied and the remaining three categories (*“Fine”*, *“Poor”*, and *“Really poor”*) were coded as less satisfied. This approach was applied in a previous study that reported a similarly high level of satisfaction [21].

Discomfort was assessed by two questionnaire items: *“Did you feel that your limits of modesty were exceeded during the examination?”* (item 33) and *“Did you feel any pain during the examination?”* (item 34). For both items, the answers were coded as yes for responses of *“To a great extent”* or *“To some extent”*, and no for responses of *“To a minor extent”* or *“Not at all”*.

**Fig. 2:**
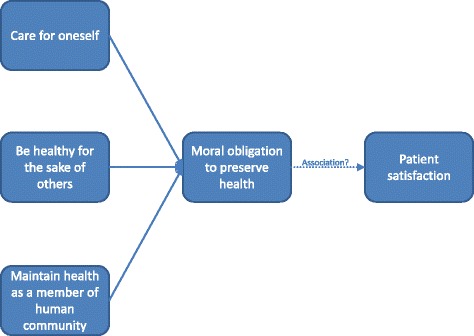
Obligations framework. Adapted from Sider et al. [22]

Based on literature research, a conceptual framework for perceived obligations in the health care system was adapted from Sider et al. [22]. It is based on the assumption that some citizens feel a moral obligation towards accepting the health preserving offers provided by the health care system. The moral obligation consists of three factors; (1) a moral obligation to preserve ones own health (instinctive obligation in order to stay alive), (2) a moral obligation to keep healthy for the sake of others (everyone has loved ones, and as such are important in the lives of others), and (3) a moral obligation to maintain healthy as a member of the human community (a mutual expectation of life-preserving behavior is expected in a human society) (Fig. 2).

Feelings of obligation were assessed by one global questionnaire item: *“Did you feel obliged to participate in the mammography screening?”* (item 6). Answers to this item were coded in the same manner as the responses for discomfort. Reasons for feeling obliged to participate (item 7) were coded as own expectations (*“the opportunity to have an eventual early detection of breast cancer in order to initiate treatment” or “I have participated in previous screenings”*), family/friends’ expectations (*“Friends/family expected me to participate”*), organizational aspects (*“To receive an invitation without having requested it”, “To receive a pre-scheduled date/time for mammography screening”, “The invitation appeared as a scheduled call for screening”*, *or “Central Denmark Region is mentioned as sender of the invitation”*), and other reasons (*“Other”*).

Screening-induced concerns were assessed by three questionnaire items concerning the three main stages in screening participation (before, during and after). Responses to *“Did you have any concerns when receiving the invitation?”* (item 8) and *“Did you feel any concern about the screening result from the time of screening until the result of screening was received?”* (item 35) were coded in the same manner as the responses for discomfort. Responses to *“After having attended the mammography screening do you experience an increased worry for developing breast cancer than before?”* (item 42) were coded as yes if the respondent answered *“More worried”* or no if the respondent answered *“Nothing has changed”* or *“Less worried”*. The three variables were combined into one category labeled screening-induced concerns, which was coded as no if all three questions were coded “no” or as yes if at least one of the three questions was coded as “yes”.

### Statistics

All statistical analyses were carried out in Stata/SE 14 (STATACorp LP, College Station, Texas, USA). Pearson’s chi-square test was used to test differences in demographic characteristic distribution between respondents and non-respondents. Test for difference in mean age between respondents and non-respondents were tested using a student’s t-test. Distributions of responses to the survey items on satisfaction, discomfort, obligations, and screening-induced concerns were calculated as proportions with 95% confidence intervals (95% CI). Proportions were used to estimate the level of screening-induced concerns among women participating in breast cancer screening. Pearson’s chi-square test was used to test for differences in proportions between women with negative vs. positive screening results.

Prevalence ratios (PR) with 95% CI were used to evaluate how satisfaction was associated with demographic characteristics, feelings of obligation to participate, discomfort, and concerns. PR was assessed using Poisson regression with robust variance to adjust for clustering between screening units. The same association measures were used to conduct sensitivity analyses omitting the women with positive screening results. The proportion of satisfied women exceeded 20%. In cross-sectional studies, logistic regression is frequently used to estimate an odds ratio (OR) as an association measure equivalent to the relative risk (RR). However, when the proportion experiencing an outcome exceeds 20%, an OR overestimates the RR, and the PR is a more accurate measure of association [23, 24].

### Power calculations

Power calculations were performed based on a 5% significance level and an 80% statistical power. To detect a difference of 72.2% vs. 81.6% in the proportions of satisfied participants between those reporting pain vs. those reporting no pain [21], we had to include at least 600 women in our study. Since there are five screening units and we expected to run cluster analyses, we had to include 3000 women in our study.

## Results

A total of 7432 women were eligible for study inclusion. Of the 3000 women randomly included, 2140 women (71.3%) completed the questionnaire (Fig. 1). Compared to non-respondents, respondents were more likely to be older, ethnic Danes, and married/cohabitating. Screening results did not differ between respondents and non-respondents (Table 1).

**Table 1:** Demographic characteristics and screening result of survey respondents and non-respondents in the survey

	Respondents (*n* = 2140)	Non-respondents (*n* = 860)	*P*-value
	*n* (%)	*n* (%)	(Chi2)
Age			
Mean	59.7	58.3	**<0.001** ^**a**^
50–54	532 (24.9)	264 (30.7)	**<0.001**
55–59	514 (24.0)	243 (28.3)	
60–64	512 (23.9)	193 (22.4)	
65–69	582 (27.2)	160 (18.6)	
Ethnicity			
Danish	2079 (97.2)	805 (93.8)	**<0.001**
Immigrant (Western country)	30 (1.4)	14 (1.6)	
Immigrant (non-western country)	29 (1.4)	39 (4.6)	
Education			
≤ 10 years	595 (28.1)	273 (32.3)	0.073
11–15 years	891 (42.1)	327 (38.7)	
> 15 years	629 (29.7)	245 (29.0)	
Marital status			
Married/Cohabitant	1740 (81.4)	658 (76.6)	**0.012**
Single	398 (18.6)	201 (23.4)	
Screening result			
Positive	44 (2.1)	18 (2.1)	0.949
Negative	2096 (97.9)	842 (97.9)	

Numbers in bold indicate statistically significant differences between respondents and non-respondents

^a^Student’s t-test for difference in means

### Overall satisfaction, discomfort, and obligations

Among the respondents, 70.3% had an excellent overall impression of the screening program and 29.4% had a fine overall impression. The telephone hot-line was rated as excellent by 79.3% of those who used it, and the web-based self-service was rated as excellent by 72.1% of the women who used it (Table 2). Among the respondents, 6.8% reported experiencing discomfort because their limits of modesty were exceeded to some or a great extent during the examination, and 17.9% reported feeling pain to some or a great extent during the examination (Table 2). A total of 36.2% of the participants reported feeling obliged to a great extent to participate in screening (Table 2). The predominantly stated reasons for feeling obliged to a great extent were in the categories of own expectations (32.8%) and organizational aspects (18.4%) (Table 2).

**Table 2:** Satisfaction, feeling obliged to participate, being concerned and feeling pain during the examination

Satisfaction	Excellent	Fine	Poor	Really poor
	% (CI)	% (CI)	% (CI)	% (CI)
What was your overall impression of the entire screening process? (*n* = 2110)	70.3 (68.3–72.2)	29.4 (27.5–31.4)	0.2 (0.1–0.5)	0.1 (0.0–0.4)
What was your overall impression of the service offered by phone? (*n* = 464)	79.3 (75.4–82.7)	20.5 (17.0–24.4)	0.0	0.2 (0.0–1.5)
What was your overall impression of the self-service facility offered? (*n* = 412)	72.1 (67.5–76.2)	24.8 (20.8–29.2)	1.9 (1.0–3.8)	1.2 (0.5–2.9)
Feeling discomfort	To a great extent	To some extent	To a minor extent	Not at all
Did you feel that your limits of modesty were exceeded…? (*n* = 2115)	3.2 (2.5–4.0)	3.6 (2.9–4.5)	6.1 (5.1–7.2)	87.2 (85.7–88.5)
Did you feel any pain during the examination? (*n* = 2129)	3.4 (2.7–4.3)	14.5 (13.1–16.1)	44.0 (41.9–46.1)	38.1 (36.1–40.2)
Feeling concerns				
Did you have any concerns when receiving the invitation? (*n* = 2113)	1.1 (0.8–1.7)	8.6 (7.5–9.9)	17.2 (15.6–18.8)	73.1 (71.1–74.9)
Did you feel any concern about the screening result from the time of screening until the result of screening was received? (*n* = 2117)	4.9 (4.1–5.9)	20.0 (18.3–21.7)	41.0 (39.0–43.2)	34.1 (32.1–36.1)
	More worried	Nothing has changed	Less worried	
After having attended the mammography screening do you experience an increased worry for developing breast cancer than before? (*n* = 2128)	2.5 (1.9–3.3)	73.0 (71.1–74.8)	24.5 (22.7–26.4)	
Feeling obliged	To a great extent	To some extent	To a minor extent	Not at all
Did you feel obliged to participate in the mammography screening? (*n* = 2087)	36.2 (34.2–38.3)	12.9 (11.5–14.4)	4.4 (3.6–5.3)	46.5 (44.4–48.7)
Reasons for feeling obliged (*n* = 1116)^a^:				
Own expectations^b^	32.8 (30.8–34.9)	10.3 (9.1–11.7)	3.3 (2.6–4.2)	No data
Family/friends expectations^b^	5.6 (4.7–6.6)	2.2 (1.7–2.9)	0.5 (0.3–0.9)	No data
Organizational aspects^b^	18.4 (16.7–20.1)	5.7 (4.8–6.8)	1.4 (1.0–2.0)	No data
Other^b^ (*n* = 63)	2.1 (1.6–2.8)	0.5 (0.3–0.9)	0.3 (0.2–0.7)	No data

^a^ *Own expectations*: Answers “The opportunity to have an eventual early detection of breast cancer in order to initiate treatment” or “I have participated in previous screenings”; *Family/friends expectations*: Answers: “Friends/family expected me to participate”; *Organizational aspects*: Answers “To receive an invitation without having requested it”, “To receive a pre-scheduled date/time for mammography screening”, “The invitation appeared as a scheduled call for screening” or “Central Denmark Region is mentioned as sender of the invitation”; *Other*: Answers “Other”

^b^Proportion of respondents reporting feeling obligated to participated due to own expectations, organizational aspects, family/friends expectations or other out of all respondents (*n* = 2087)

### Screening-induced concerns

Among the respondents, 72.6% reported no screening-induced concerns, including 73.3% of the women with negative screening results and 38.1% of women with positive screening results. Some women reported experiencing concerns both when receiving the invitation and while waiting for the results, and were more concerned about breast cancer after their screening—including 0.8% of women with negative screening results and 11.9% of women with positive screening results (Table 3). The largest proportions of women reported concerns while awaiting their results: 24.1% of those with negative screening results, 50.0% of those with positive screening results; (*p* < .001).

**Table 3:** Screening induced concerns in women participating in breast cancer screening

	All (*n* = 2083)	Neg. screening (*n* = 2041)	Pos. screening (*n* = 42)
	% (95% CI)	% (95% CI)	% (95% CI)
Reported no screening induced concerns^a^	72.6 (70.6–74.5)	73.3 (71.3–75.2)	38.1 (24.3–54.1)
Concerns induced “only” at one point	18.8 (17.2–20.6)	18.4 (16.8–20.2)	40.5 (26.3–56.4)
At the receiving of the invitation	*1.9 (1.4–2.6)*	*2.0 (1.4–2.7)*	*0.0 (−)*
While waiting for the result	*16.3 (14.8–18.0)*	*16.0 (14.5–17.7)*	*28.6 (16.6–44.6)*
More concerned after participation	*0.6 (0.3–1.0)*	*0.3 (0.2–0.7)*	*11.9 (4.9–26.4)*
Concerns induced twice	7.5 (6.5–8.8)	7.5 (6.4–8.7)	9.5 (3.5–23.6)
At the receiving of the invitation *and* while waiting for the result	*6.6 (5.6–7.7)*	*6.6 (5.6–7,7)*	*7.1 (2.2–20.7)*
At the receiving of the invitation *and* more concerned after participation	*0.2 (0.1–0.5)*	*0.2 (0.1–0.5)*	*0.0 (−)*
While waiting for the result *and* more concerned after participation	*0.8 (0.5–1.3)*	*0.7 (0.4–1.2)*	*2.4 (0.3–16.2)*
Screening induced concerns at all points	1.1 (0.7–1.6)	0.8 (0.5–1.3)	11.9 (4.9–26.3)

Numbers in *italic* are subdivisions of the above non italic proportions

Pearson’s chi-square test for difference between level of concern in women with positive screening test vs. negative screening test was <0.001 for all items

^a^No screening induced concerns refers to all women answering “To a minor extent” or “Not at all” in items on concerns at the receiving of the invitation and while waiting for the result and answering “Nothing has changed” or “Less worried” in the item on concerns after participation

### Factors associated with satisfaction

Analyses of associations between satisfaction with the screening program and demographic characteristics revealed that compared to women of 50–54 years of age older women showed slightly lower satisfaction: adjusted PR_55–59_, 0.94 (95% CI, 0.90–0.99); adjusted PR_60–64_, 0.92 (95% CI, 0.87–0.97); adjusted PR_65–70_, 0.94 (95% CI, 0.86–1.04). Additionally, immigrants from non-western countries showed lower satisfaction than ethnic Danes: adjusted PR, 0.75 (95% CI, 0.58–0.97) (Table 4). Overall, women who experienced discomfort showed lower satisfaction. Women who experienced pain showed lower satisfaction than women without pain: adjusted PR, 0.82 (95% CI, 0.74–0.91). Likewise, women who felt that their limits of modesty were transgressed during the examination were less satisfied than women who did not have this experience: adjusted PR, 0.79 (95% CI, 0.71–0.88). Similarly, women who felt obliged to participate reported lower satisfaction than women without that feeling: adjusted PR, 0.96 (95% CI, 0.92–0.99). Being concerned at any time during the screening process was also associated with lower satisfaction (PR, 0.84; 95% CI, 0.77–0.91), whereas screening result was not associated with satisfaction (Table 4).

**Table 4:** Associations between satisfaction and background data and remaining outcomes

	Unadjusted PR (CI)	Adjusted^a^ PR (CI)
Age		
50–54 years	1 (ref)	1 (ref)
55–59 years	**0.93 (0.89–0.98)** ^‡^	**0.94 (0.90–0.99)** ^‡^
60–64 years	**0.93 (0.88–0.98)** ^‡^	**0.92 (0.87–0.97)** ^‡^
65–70 years	0.94 (0.86–1.03)	0.94 (0.86–1.04)
Marital status		
Married/Cohabitant	1 (ref)	1 (ref)
Single	1.03 (0.96–1.11)	1.03 (0.96–1.11)
Ethnicity		
Danish	1 (ref)	1 (ref)
Immigrant (Western)	1.08 (0.91–1.27)	1.03 (0.85–1.25)
Immigrant (non-western)	**0.71 (0.56–0.90)** ^‡^	**0.75 (0.58–0.97)** ^‡^
Education		
≤ 10 years	**0.92 (0.89–0.96)**	**0.93 (0.90–0.96)**
11–15 years	1 (ref)	1 (ref)
> 15 years	0.96 (0.92–1.01)	0.96 (0.91–1.01)
Discomfort		
Pain (2)		
No	1 (ref)	1 (ref)
Yes	**0.83 (0.75–0.92)** ^‡^	**0.82 (0.74–0.91)** ^‡^
Limits of modesty exceeded (2)		
No	1 (ref)	1 (ref)
Yes	**0.79 (0.70–0.89)**	**0.79 (0.71–0.88)**
Feeling obliged (2)		
No	1 (ref)	1 (ref)
Yes	**0.94 (0.91–0.98)** ^‡^	**0.96 (0.92–0.99)** ^‡^
Being concerned		
At invitation (2)		
No	1 (ref)	1 (ref)
Yes	**0.66 (0.51–0.86)** ^‡^	**0.67 (0.52–0.85)** ^‡^
Waiting for result(2)		
No	1 (ref)	1 (ref)
Yes	**0.84 (0.77–0.92)** ^‡^	**0.85 (0.78–0.93)** ^‡^
More concerns after screening (3)		
No	1 (ref)	1 (ref)
Yes	**0.71 (0.50–0.99)** ^‡^	**0.70 (0.50–1.00)** ^‡^
Concerns at any time		
No	1 (ref)	1 (ref)
Yes	**0.83 (0.76–0.91)**	**0.84 (0.77–0.91)**
Screening test		
Positive	1 (ref)	1 (ref)
Negative	1.04 (0.87–1.26)	1.02 (0.83–1.26)

Associations between socio-demography, discomfort, obligations, concerns and being highly satisfied with breast cancer screening (1)

Prevalence Ratio (PR) with 95% confidence interval (CI)

Numbers in bold indicate statistically significant results

^‡^ *P*-value <0.05

^a^Adjusted for age, marital status, education and ethnicity

(1) Highly satisfied is defined as women answering “Excellent” in the question “What was your overall impression of the entire mammography screening process?” Less satisfied is defined as women answering either “Fine”, “Poor”, or “Really poor” in the same question

(2) Yes is defined as women answering either “to a great extent” or “to some extent”, while No is defined as women answering “to a minor extent” or “not at all”

(3) Yes is defined as women answering “more worried”, while No is defined as women answering “nothing has changed” or “less worried”

## Discussion

### Main findings

The results of this cross-sectional study revealed that almost all participants were satisfied with the breast cancer screening program. However, satisfaction was lower among women who felt discomfort during the screening examination, felt obliged to participate, or experienced screening-induced concerns. Lower satisfaction was also reported by older women, non-western immigrants, and women with low education levels.

The level of satisfaction was not influenced by screening results. Women with positive screening results generally reported more screening-induced concerns than women with negative screening results. However, concerns that arose while waiting for the results were most commonly reported, regardless of the screening result. Surprisingly, almost two out of five women with positive screening results reported no screening-induced concerns.

### Strengths and limitations

One strength of this study is that participants were invited from the complete list of women receiving the results of their breast cancer screening examination during the study period. Thus, the women who were randomly selected from the five screening units are highly representative of the target population. Additionally, the data regarding demographic characteristics had very few missing values due to the completeness of the Danish CRN system. One potential problem could be that the data from Statistics Denmark was from 2012, while the survey was conducted in 2013. However, ethnicity classification cannot change between years, and changes in education and marital status are expected to be negligible. Only 0.6% of women in this age group were getting divorced in 2013 [25]. Thus, the retrieved demographic data are expected to be very valid and of high quality. Our study benefitted from the opportunity to adjust for confounding of parameters obtained from high-validity registers.

With regards to the data collected by questionnaire, selection bias is one potential weakness. The survey response rate was fairly high (71.3%), but the respondents and the non-respondents significantly differed in age, ethnicity, and marital status. The groups that were best represented in the survey (the oldest age groups, Danish women, and married/cohabitating women) tended to show higher satisfaction than other groups, suggesting that the general satisfaction in the population may be lower than the overall rate in our study. Furthermore, all information was collected retrospectively, opening up the possibility of recall bias, which may differ between women with negative and positive screening results. However, sensitivity analyses showed that omitting women with positive screening results did not alter the associations. Recall bias may also especially apply to questions about emotions related to the screening invitation that was received up to 2 months earlier.

Finally, as this study was embedded within the ongoing quality assurance program in our region, we did not measure satisfaction or concerns using existing scales. However, all questions were chosen based on literature review, and subsequently underwent an ad hoc procedure within the target population to test for face-validity, as described. Overall, we believe that this study has adequate internal validity, and that the results can be generalized at least to women participating in the Danish breast cancer screening program, and likely also to similarly managed programs in other countries.

### Interpretation of results

Previous studies in countries with free population-based breast cancer screening programs have reported high levels of general satisfaction [21, 26, 27]. Compared with a previous satisfaction survey in the CDR in 2010 [28], our present results showed an even higher level of general satisfaction. This is somewhat surprising as the process in the CDR has been highly streamlined, with only 5 min allocated to each examination. Our findings indicate that women accept this effective organization, or may even prefer it over a more time-consuming procedure. However, the efficiency of this procedure may be at the expense of personal considerations for the women’s modesty, causing some women to be less satisfied.

Although satisfaction was very high in this study, we observed a U-shaped tendency according to educational level—with lower satisfaction among women with lower or higher educational levels and greater satisfaction among those with a medium educational level. Jensen et al. observed a similar association between educational level and screening participation in the CDR—with low-educational and high-educational women showing PR of nonparticipation of 1.1 and 1.15, respectively [29]. Together, these findings suggest that medium-educated women participate more and are more satisfied with the screening program compared to other women in the CDR. Studies on information needs and general perception of the screening offer in lower and higher educational level citizens are needed in order to know how to reach the different sub-groups in a screening setting.

In previous studies, pain during screening has been reported by 8.8% [30] and 6% [31] of women, which is less than the 18% observed in our study. This difference could be due to different methods of measurement, which cannot be compared due to limited information in the publications. In our study, pain during screening was associated with lower degree of satisfaction. Accordingly, Almog et al. previously reported a RR of 1.5 for being satisfied among participants who experienced no discomfort compared to those feeling the most discomfort [21]. Importantly, improvements in techniques should focus on ensuring little or no pain in the breast cancer screening experience, without compromising mammogram quality.

To our knowledge, no prior study has estimated how many women feel obliged to participate in breast cancer screening, or whether feeling obliged to participate is associated with satisfaction or continued participation. Here we found that half of the participants felt obliged to some or a great extent to participate in breast cancer screening, mainly for reasons categorized as their own expectations or organizational aspects. It has been argued that most people are authoritarianists and want to preserve their health, and would therefore feel guilty about not participating in an examination aimed at preserving ones health when invited by letter with a pre-booked appointment [13]. Our results indicated that women who felt obliged to participate were primarily driven by their wish to preserve their health and to a lesser extend by the fact that the invitation included a pre-booked appointment. This suggests that it is not as much the organizational aspects of the screening program as it is the offer to participate itself that makes women feel obliged to participate, and it could be that the main pressure comes from a wish to preserve health with the pre-booked appointment only adding up on that feeling. Women who felt obliged to participate were slightly less satisfied than women who did not feel obliged to participate. Since patient satisfaction is based on different factors of perceived quality [20] it could also be hypothesized that a citizen taking up screening because of obligations to others (family/friends/society) might experience lower levels of satisfaction. However, further research is needed to examine why and how some women feel obliged to participate and how it affects them and their experience with breast cancer screening.

A New Zealand study reported that 11% of women participating in breast cancer screening were worried while waiting for the screening examination, 18% while waiting for the results, and 1% were more worried after completing the screening [32]. Women in our study experienced somewhat more screening-induced concerns than women in the New Zealand study, but the tendencies were similar. Women reported that they predominantly experienced concerns while waiting for their results, underlining the importance of quickly delivering the screening results to participating women to minimize their concerns.

In a previous study, a substantial proportion of women with false-positive screening results experienced mammography-related anxiety (47%) or cancer-related worries (41%) 3 months later; however, these worries did not influence subsequent screening adherence [33]. Another study reported that compared to women with normal findings, those with false-positive screening results consistently experienced greater negative psychological consequences at 3 months and 3 years later [34]. Our study was not designed to measure long-term consequences of false-positive screening results. However, the previously reported figures seem rather large compared to our present finding that 25.6% of women with a positive screening result experienced more concerns a few weeks after participating in the screening compared to before the screening. Additional research could help to fully understand the extent of concerns and anxiety among women participating in breast cancer screening.

Peipins et al. found that satisfied women tend to paticipate more in future screening examinations [9]. We did not explore this in our study, but we do know from a previous satisfaction survey in our region, that the general satisfaction is also high 3 years earlier [28]. Thus high continuing satisfaction may be one reason for a continuing high participation rate above 80% in our region.

## Conclusions

Overall, satisfaction was very high among women participating in breast cancer screening in the CDR. Lower satisfaction was reported among women who experienced discomfort, obligations, or concerns and among non-western immigrants. The present results indicate that concerns regarding breast cancer were introduced only among a minimum of participating women with normal screening results, and to a lesser degree in women with positive screening results as compared to previous studies. Further, almost half of the women stated that they felt obliged to participate, which calls for further research in order to understand its significance and implications.

It appears that a continuing focus on high service in breast cancer screening is important for achieving the highest benefit from the program. This includes initiatives to employ the least painful techniques, to respect the patients’ modesty as much as possible, to deliver fast screening results and thus minimize concerns among women awaiting results, and to design interventions with different minorities in mind.

## Additional file

Additional file 1: Questionnaire. (PDF 107 kb)

## Abbreviations

CDR: Central Denmark Region
CI: Confidence interval
CRN: Civil registration number
OR: Odds ratio
PR: Prevalence ratio
RR: Relative risk
