# Supplementary material for: Satisfaction, discomfort, obligations, and concerns in population-based breast cancer screening: cross-sectional study in a Danish population
Source: BMC Health Serv Res. 2017 Jul 14;17:489. doi: 10.1186/s12913-017-2438-2 (PMC5513139; doi:10.1186/s12913-017-2438-2)
Supplement: Additional file 1: — Questionnaire. (PDF 107 kb) [file 12913_2017_2438_MOESM1_ESM.pdf]

Regionshuset
Århus
Center for Kvalitetsudvikling
Olof Palmes Allé 15
8200 Århus N
www.regionmidtjylland.dk

You have recently participated in a population-based mammography screening initiated by Department of Public Health Programmes, Central Denmark Region

midt
regionmidtjylland

In order to be able to improve the invitation to mammography screening and the information provided with this invitation, Department of Public Health Programmes is interested in learning about **your experience** with the entire process of participation.

Therefore, we allow ourselves to make this approach and to kindly ask you to fill in the attached questionnaire. Having completed the questionnaire, please return it in the pre-paid postage envelope. You may also fill in the questionnaire through www.svar.rm.dk by using the code *>> code >>.* Please observe the deadline *>> date >>.*

When responding to the questions we ask you to focus on your experience with the screening conducted in 20xx, although you may have participated in previous screenings.

CFK – Folkesundhed og Kvalitetsudvikling (Public Health and Quality Development) has been assigned to assess the invitation to mammography screening on behalf of Department of Public Health Programmes, Central Denmark Region.

CFK – Folkesundhed og Kvalitetsudvikling (Public Health and Quality Development) will ensure that your response will be treated confidentially and all responses will appear anonymously in the final report. The report will be available in *month/year* on the website of Central Denmark Region www.rm.dk.

For any questions relating to the questionnaire, please contact CFK – Folkesundhed og Kvalitetsudvikling (Public Health and Quality Development) consultant Dorte Jørgensen, phone no. +45 7841 4037/Dorte.Jorgensen@stab.rm.dk.

Best regards,
Department of Public Health Programmes
Central Denmark Region

Invitation

Question	Response options
1. Were you aware of the mammography screening programme prior to receiving the invitation?	☐ Yes ☐ No ☐ I don't know
2. Did you find it easy to understand the invitation?	☐ To a great extent ☐ To some extent ☐ To a minor extent ☐ Not at all ☐ I don't know
3. Did the invitation include the information you needed prior to screening?	☐ To a great extent ☐ To some extent ☐ To a minor extent ☐ Not at all ☐ I don't know
4. Have you searched for additional information about mammography screening?	☐ No<br>☐ Yes, please mention where information has been searched<br>____________________________________
5. Was it easy for you to decide whether to participate in the mammography screening?	☐ To a great extent ☐ To some extent ☐ To a minor extent ☐ Not at all ☐ I don't know
6. Did you feel obliged to participate in the mammography screening?	☐ To a great extent ☐ To some extent ☐ To a minor extent ☐ Not at all **Please go to question no. 8** ☐ I don't know
7. What made you feel obliged? *(Several responses can be chosen)*	☐ To receive an invitation without having requested it<br>☐ To receive a pre-scheduled date/time for mammography screening<br>☐ The opportunity to have an eventual early detection of breast cancer in order to initiate treatment<br>☐ I have participated in previous screenings<br>☐ The invitation appeared as a scheduled call for screening<br>☐ Friends/family expected me to participate<br>☐ Central Denmark Region is mentioned as sender of the invitation<br>☐ Other, please list: ________________________
8. Did you have any concerns when receiving the invitation?	☐ To a great extent ☐ To some extent ☐ To a minor extent ☐ Not at all ☐ I don't know
9. Was the period from receiving the invitation until scheduled appointment for screening suitable?	☐ Yes ☐ No, too short ☐ No, too long ☐ I don't know

Self-service (through www.kraeftscreening.dk)

Question	Response options
10. Did you change your appointment or venue for screening through self-service?	☐ Yes ☐ No **Please go to question no. 17**
11. Did you find it easy to use the website?	☐ To a great extent ☐ To some extent ☐ To a minor extent ☐ Not at all ☐ I don't know
12. **When using the self-service to alter the appointment:** Were you satisfied with the possibilities to choose another date/time?	☐ To a great extent ☐ To some extent ☐ To a minor extent ☐ Not at all ☐ I don't know /Not applicable
13. **When using the self-service to alter the screening venue:** Were you satisfied with the possibilities to choose another screening venue?	☐ To a great extent ☐ To some extent ☐ To a minor extent ☐ Not at all ☐ I don't know/Not applicable
14. Did you solve your problem by using the self-service?	☐ To a great extent ☐ To some extent ☐ To a minor extent ☐ Not at all ☐ I don't know

15. What is your overall impression of the self-service facility offered?	Excellent ☐	Fine ☐	Poor ☐	Really poor ☐	I don't know ☐
16. Can you suggest any improvements in order to optimize the self-service facility?					

Contact by phone to Department of Publich Health Programmes prior to screening

17. Have you been in contact with Department of Public Health Programmes by phone prior to your mammography screening? (e.g. in order to arrange a rescheduling of time/venue)	Yes ☐		No ☐ **Please go to question no. 26**		
18. How did you experience the phone queue when calling Department of Public Health Programmes?	Acceptable ☐		Unacceptable ☐		No queue ☐
19. Were you satisfied with the phone opening hours through which it was possible to call Department of Public Health Programmes?	To a great extent ☐	To some extent ☐	To a minor extent ☐	Not at all ☐	I don't know ☐

20. Why did you call Department of Public Health Programmes (Several responses can be chosen)

- ☐ Rescheduling of date/time
- ☐ Alteration of screening venue
- ☐ Questions regarding screening
- ☐ Registration for screening
- ☐ Other, please state:

21. Was your call answered professionally?	To a great extent ☐	To some extent ☐	To a minor extent ☐	Not at all ☐	I don't know ☐
22. **If calling in order to alter the date/time for screening:** Were you satisfied with the possibilities to choose a new date/time?	To a great extent ☐	To some extent ☐	To a minor extent ☐	Not at all ☐	I don't know/not applicable ☐
23. **If calling in order to alter screening venue?** Were you satisfied with the possibil-ities to choose another screening venue?	To a great extent ☐	To some extent ☐	To a minor extent ☐	Not at all ☐	I don't know/not applicable ☐
24. **If calling in order to clarify question(s) regarding the screening/examination:** Were you satisfied with the respons-es/information you received?	To a great extent ☐	To some extent ☐	To a minor extent ☐	Not at all ☐	I don't know/not applicable ☐
25. What is your overall impression of the ser-vice offered by phone?	Excellent ☐	Fine ☐	Poor ☐	Very poor ☐	I don't know ☐

Examination at the screening venue (Health Center/Hospital)

26. Were the parking lots at the screening ven-ue satisfactory?	To a great extent ☐	To some extent ☐	To a minor extent ☐	Not at all ☐	I don't know ☐	Not appli-cable ☐
27. How did you experience the welcome when entering the health center/hospital?	Excellent ☐	Fine ☐	Poor ☐	Really poor ☐	I don't know ☐	
28. Did you experience excessive waiting in the waiting room passing your scheduled ap-pointment time?	No wait ☐	Less than 5 min. ☐	5-10 min. ☐	11-15 min. ☐	More than 15 min. ☐	
29. Did the staff handle the examination profes-sionally?	To a great extent ☐	To some extent ☐	To a minor extent ☐	Not at all ☐	I don't know ☐	

30. Did you feel comfortable in the way which the staff conducted the examination?	To a great extent ☐	To some extent ☐	To a minor extent ☐	Not at all ☐	I don't know ☐	
31. Did the staff listen to your questions/comments during the examination?	To a great extent ☐	To some extent ☐	To a minor extent ☐	Not at all ☐	I don't know ☐	Not applicable ☐
32. Did the staff provide sufficient information during the examination?	To a great extent ☐	To some extent ☐	To a minor extent ☐	Not at all ☐	I don't know ☐	Not applicable ☐
33. Did you feel that your limits of modesty were exceeded during the examination?	To a great extent ☐	To some extent ☐	To a minor extent ☐	Not at all ☐	I don't know ☐	
34. Did you feel any pain during the examination?	To a great extent ☐	To some extent ☐	To a minor extent ☐	Not at all ☐	I don't know ☐	

Result of screening

35. Did you feel any concern about the screening result from the time of screening until the result of screening was received?	To a great extent ☐	To some extent ☐	To a minor extent ☐	Not at all ☐	I don't know ☐	
36. Did you find it easy to understand the response letter?	To a great extent ☐	To some extent ☐	To a minor extent ☐	Not at all ☐	I don't know ☐	
37. Did the response letter include all necessary information?	To a great extent ☐	To some extent ☐	To a minor extent ☐	Not at all ☐	I don't know ☐	
38. Was the period from screening until you received the response letter suitable?	Yes ☐ **Please go to question no. 40**	No, too short ☐	No, too long ☐		I don't know ☐	
39. **If the period from screening until you received the response letter was NOT suitable:** Did you have any concerns?	To a great extent ☐	To some extent ☐	To a minor extent ☐	Not at all ☐	I don't know ☐	Not applicable ☐

Overall impression of the entire screening process

40. What is your overall impression of being invited to attend mammography screening?	Excellent ☐	Fine ☐	Poor ☐	Really poor ☐	I don't know ☐	
41. What is your overall impression of the entire mammography screening process (from receiving the invitation until response letter was received)?	Excellent ☐	Fine ☐	Poor ☐	Really poor ☐	I don't know ☐	
42. After having attended the mammography screening do you experience an increased worry for developing breast cancer than before?	More worried ☐		Nothing has changed ☐		Less worried ☐	
43. Do you intend to accept the next invitation for mammography screening?	Yes ☐		No ☐		I don't know ☐	Not applicable ☐
44. Do any of your relatives or closest friends have cancer?	☐ No<br>☐ Yes, breast cancer<br>☐ Yes, which type of cancer: ______					

45. Can you suggest any improvements in order to optimize the screening process for breast cancer?	
46. Would you choose the opportunity to receive a reminder text message if this becomes an option?	Yes ☐ No ☐ I don't know ☐ Not applicable ☐

Your background

47. Do you attend a job at present?	Yes, I attend a job ☐ No, I am retired/ early retirement ☐ No, I do not attend at job at present ☐
48. What is your highest level of education? (Irrespective whether you have retired/made early retirement)	Primary school ☐ High school/college or similar ☐ Skilled worker (craftsman, within trade or office) ☐ Short-cycle higher education ☐ Medium-cycle higher education ☐ Long-cycle higher education ☐
49. Have you had a mammography screening on previous occasion?	Yes ☐ No ☐ I don't know ☐
50. Please state the number of kilometers from your private address to the screening venue	☐☐☐ kilometers

CFK – Folkesundhed og Kvalitetsudvikling - 2013
